# Development of Incompletely Fused Carpels in Maize Ovary Revealed by miRNA, Target Gene and Phytohormone Analysis

**DOI:** 10.3389/fpls.2017.00463

**Published:** 2017-04-03

**Authors:** Hongping Li, Ting Peng, Qun Wang, Yufeng Wu, Jianfeng Chang, Moubiao Zhang, Guiliang Tang, Chaohai Li

**Affiliations:** ^1^Collaborative Innovation Center of Henan Grain Crops, Agronomy College, Henan Agricultural UniversityZhengzhou, China; ^2^National Key Laboratory of Crop Genetics and Germplasm Enhancement, Bioinformatics Center, Nanjing Agricultural UniversityNanjing, China

**Keywords:** maize, incomplete carpel fusion, ovary development, miRNA, degradome, phytohormone

## Abstract

Although the molecular basis of carpel fusion in maize ovary development remains largely unknown, increasing evidence suggests a critical role of microRNAs (miRNAs). In this study, a combination of miRNA sequencing, degradome and physiological analyses was used to characterize carpel fusion development in maize ovaries showing incompletely (IFC) and completely fused carpels (CFC). A total of 162 known miRNAs distributed across 33 families were identified, of which 20 were differentially expressed. In addition, 53 miRNA candidates were identified, of which 10 were differentially expressed in the IFC and CFC ovaries. In degradome analysis, a total of 113 and 11 target genes were predicted for the known and novel miRNAs, respectively. Moreover, 24 (60%) target genes of the differentially expressed known miRNAs were found to code transcription factors, including auxin response factor (ARF), TB1-CYC-PCFs (TCP), APETALA2 (AP2), growth regulating factor (GRF), MYB, NAC, and NF-YA, all of which have been shown to play a role in carpel fusion development. Correlation analysis of these differentially expressed known miRNAs and their targets with phytohormone signals revealed significant correlations with at least one phytohormone signal, the main regulator of carpel fusion development. These results suggest that incomplete carpel fusion is partly the result of differential expression of certain miRNAs and their targets. Overall, these findings improve our knowledge of the effect of miRNA regulation on target expression, providing a useful resource for further analysis of the interactions between miRNAs, target genes and phytohormones during carpel fusion development in maize.

## Introduction

Maize (*Zea mays L*.) is one of the most productive cereals in the world, and is widely used as a model organism for genetic research in cultivated crop plants (Wallace et al., 2014). Maize is monocotyledon, with two different types of inflorescence in a single plant: the tassel (male inflorescence) and the ear (female inflorescence) (McSteen et al., 2000). Development of the pistil in female inflorescences is crucial to maize kernel development and grain yield formation. The inner whorl of the floret, the maize pistil is derived from fusion of three carpels, and is composed of a silk and an ovary (McSteen et al., 2000). Incomplete carpel fusion results in a hole in the pericarp of the kernel. Without the protection of the intact pericarp, the kernel is highly susceptible to pathogen infection, threatening food safety (Duncan and Howard, 2010). In addition, these kernels rot more easily after sowing, and thus, complete carpel fusion is essential for food security as well as seed vigor quality. However, despite this, few studies have examined carpel fusion development in maize. Although several genes associated with carpel organ identity have been identified in maize using reverse genetics and molecular studies (Mena et al., 1996), knowledge of carpel fusion remains limited and regulation of the genes involved is unknown.

Incomplete carpel fusion is thought to be regulated by transcription factors (TFs) and hormonal balance (Reyes-Olalde et al., 2013; Marsch-Martínez and de Folter, 2016). Mutations in TFs such as APETALA2 (AP2), CUP-SHAPED COTYLEDON2 (CUC2) and SPATULA (SPT) can lead to partially or almost completely unfused carpels in the gynoecium of *Arabidopsis* (Ripoll et al., 2011; Nahar et al., 2012). The *Arabidopsis* mutant *HECATE* has a low auxin (IAA) content and presents a carpel fusion-deficient phenotype in the gynoecium (Schuster et al., 2015). In line with this, a high concentration of IAA was found to be important for apical fusion of the two carpels in the stigma of the *Arabidopsis* gynoecium (Larsson et al., 2013; Sehra and Franks, 2015). Moreover, plant hormones are known to affect transcriptional regulation through hormone sensing, synthesis and transport (Marsch-Martínez and de Folter, 2016). To understand carpel fusion in maize, the mechanisms regulating carpel fusion and limiting intact ovary wall formation need to be determined, based not only on genetic research and conventional physiological studies, but also at the level of post-transcriptional regulation.

MicroRNAs (miRNAs) are small non-coding RNA molecules that negatively regulate gene expression mainly through mRNA cleavage or translational inhibition, or DNA methylation of miRNA genes (Voinnet, 2009). MiRNAs, which are generated from single-strand RNA precursors able to form hairpin structures, have been widely studied as essential regulators of diverse aspects of plant development (Larue et al., 2009), including flower development. For example, maize *ts4*, which encodes miR172 and targets the *AP2* homolog *indeterminate spikelet 1*, has two tandem AP2 domains and plays an important role in regulating maize inflorescence development (Chuck et al., 2007). In *ts4* mutants, the floret of the male inflorescence fails to form stamens and develops unfused carpels (Chuck et al., 2007), suggesting a role of miR172 in regulation of carpel fusion development in maize female inflorescences. Furthermore, in *Arabidopsis*, miR164 has been shown to target a subset of NAC TFs that includes CUC1 and CUC2, which contribute to organ boundary formation including carpel marginal tissue development (Nahar et al., 2012). Moreover, miR160, which is believed to target mRNA coding ARF DNA-binding protein, is thought to be involved in female and male flower development in poplar through regulation of auxin signaling (Song et al., 2013). Thus, miRNAs have great potential as a tool for elucidating floral organ development in maize and other plant species.

In this study, we used high-throughput sequencing to detect miRNA activity at the moment of incomplete carpel fusion could being morphological distinguished, and identified their targets through degradome analysis, which was previously used to identify miRNA-mRNA target pairs in tomato, maize and grapevine (Pantaleo et al., 2010; Karlova et al., 2013; Liu et al., 2014). Subsequently, the results were validated by quantitative RT-PCR (qRT-PCR) analysis of ovary formation and growth development in IFC and CFC ovaries. In addition, contents of six phytohormones during ovary development, prior to pollination, were determined as well. Data showing the interactions between differentially expressed miRNAs, target genes and phytohormone signals will contribute to our understanding of the molecular foundation of carpel fusion during maize ovary development.

## Results

### Phenotypic differences between IFC and CFC ovaries and kernels

**Figure 1:**
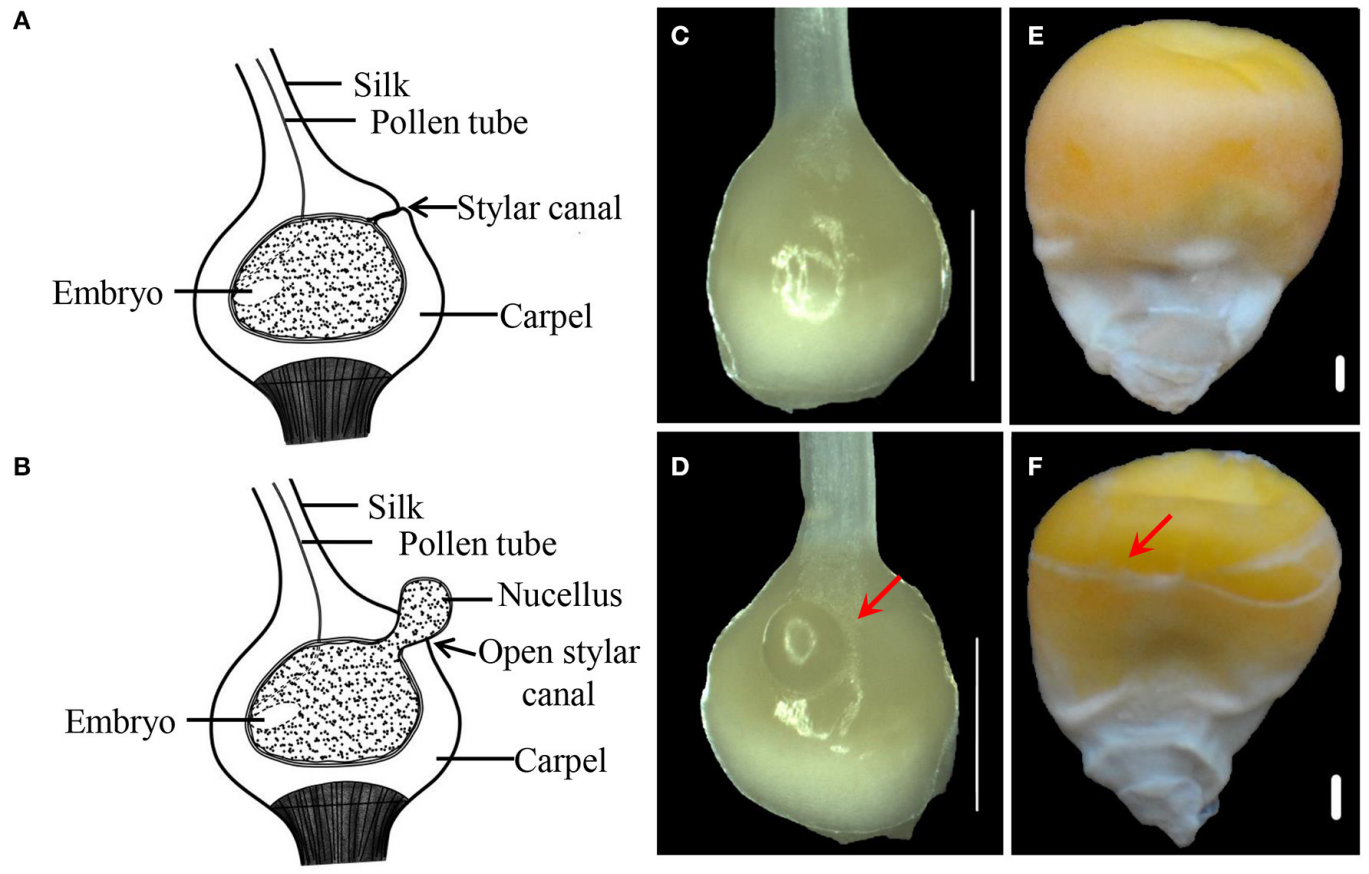
**Phenotypes of IFC and CFC ovaries and kernels at different stages of maize kernel development. (A,B)**, longitudinal section through a normal pistil and an IFC pistil, respectively. **(C,D)**, CFC and IFC ovaries at the silking stage, respectively. The extruding nucellus is indicated by an arrow in the IFC ovary. **(E,F)**, mature kernels of CFC and IFC ovaries with whole and incomplete pericarps, respectively. Bars = 1 mm. IFC, incompletely fused carpels; CFC, completely fused carpels.

Compared with CFC ovaries, IFC ovaries showed an incomplete carpel wall with a hole in the top, near the silk. Moreover, with growth and expansion, the inner tissue (nucellus) extruded from the carpel wall in the IFC ovaries (Figures 1A–D). After pollination, IFC kernels lacking complete pericarp wrapping became easily deformed on contact with other kernels, resulting in an irregular shape compared to CFC kernels at maturity (Figures 1E,F).

### Global analysis of small RNAs in IFC and CFC ovaries

**Figure 2:**
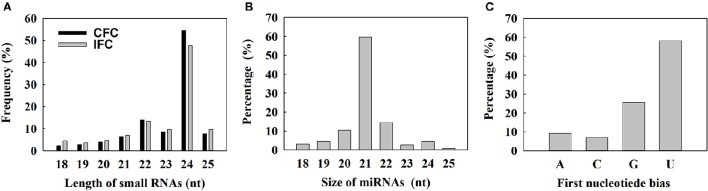
**Size distribution of small RNAs and characterization of known miRNAs detected by deep sequencing. (A)** Length distribution of the sequenced small RNAs; **(B)** Distribution of obtained unique known miRNAs; **(C)** Percentage of first nucleotide bias in the identified unique known miRNAs.

To determine the involvement of regulatory miRNAs in carpel fusion during maize ovary formation, we profiled miRNA variation in IFC and CFC ovaries. An average of 11,762,937 and 11,545,117 raw reads were obtained from IFC and CFC ovaries, respectively, which after filtering, represented an average of 6,049,566 valid reads representing 2,658,536 unique sequences and 7,085,972 valid reads representing 3,565,628 unique sequences, respectively (Table 1). These unique sequences were subsequently used to identify known and novel miRNAs by alignment against miRBase (Version 21.0). The length of the sRNAs ranged from 18 to 25 nt, and in both the IFC and CFC libraries, the 24 nt category was most abundant (average of 51.07%; Figure 2A). This is consistent with the typical lengths of plant sRNAs reported in other studies (Liu et al., 2014; Hackenberg et al., 2015). Of the known miRNAs, 21-nt miRNAs were most abundant (59.7%) (Figure 2B), representing the dominant size of mature miRNAs in plants.

**Table 1:** **Distribution of small RNAs in different categories**.

**Category**	**IFC**		**CFC**	
	**Total sRNAs**	**Unique sRNAs**	**Total sRNAs**	**Unique sRNAs**
Raw reads	11,762,937 (100%)	3,991,559 (100%)	11,545,117 (100%)	4,727,969 (100%)
Valid reads	6,049,566 (51.43%)	2,658,536 (66.60%)	7,085,972 (61.38%)	3,565,628 (75.42%)
3ADT&length filter	4,180,440 (35.54%)	979,900 (24.55%)	2,768,896 (23.98%)	748,476 (15.83%)
Junk reads	52,382 (0.45%)	40,189 (1.01%)	59,737 (0.52%)	44,287 (0.94%)
Rfam	958,537 (8.15%)	42,949 (1.08%)	1,008,711 (8.74%)	44,726 (0.95%)
mRNA	527,963 (4.49%)	271,750 (6.81%)	627,869 (5.44%)	326,516 (6.91%)
Repeats	20,960 (0.18%)	1,969 (0.05%)	19,358 (0.17%)	2,178 (0.05%)
rRNA	660,782 (5.62%)	24,733 (0.62%)	614,595 (5.32%)	25,627 (0.54%)
tRNA	229,531 (1.95%)	8,623 (0.22%)	330,777 (2.87%)	9,214 (0.19%)
snoRNA	15,218 (0.13%)	2,860 (0.07%)	12,862 (0.11%)	2,791 (0.06%)
snRNA	6,938 (0.06%)	2,893 (0.07%)	7,410 (0.06%)	2,977 (0.06%)
Other Rfam RNA	46,070 (0.39%)	3,841 (0.10%)	43,068 (0.37%)	4,117 (0.09%)

*IFC, incompletely fused carpels; CFC, completely fused carpels; 3ADT, 3′ adaptor*.

The 5′ terminal nucleotides of sRNA sequences influence classification of their AGO complexes and is an important feature affecting function. Most miRNAs are incorporated into the AGO1 effector complex, resulting in sequence specificity that either cleaves or translationally represses their targets (Rogers and Chen, 2013). AGO1 harbors miRNAs that favor the 5′ terminal uridine (Mi et al., 2008); therefore, we examined the 5′ nucleotide distribution of known miRNAs obtained from IFC and CFC ovaries. Of 172 mature miRNA sequences, 58.15% started with uridine at the 5′-end, and 25.56% started with Guanine (Figure 2C). We also examined the sequence frequency and distribution of known and novel miRNA candidates across precursor sequences in the two libraries (Supplementary Table S1). Similar to other deep sequencing studies (Peng et al., 2011; Tian et al., 2011), unique miRNAs outnumbered unique miRNA precursors, suggesting that two or more mature miRNAs are distributed in different parts of the same precursor body, their expression levels determining the dominant miRNA.

### Differentially expressed miRNAs between IFC and CFC ovaries

miRNAs have important functions in plant development and stress responses, and are a well-studied class of regulatory sRNAs. To determine differential expression between IFC and CFC ovaries, miRNA expression was normalized to transcripts per 1,000,000 and simplified as normalized expression (NE). In a given sample, a miRNA was considered if the NE value was greater than one for a known miRNA. Based on this criterion, a total of 162 (50.47% of the total) mature known miRNAs belonging to 33 families were observed in at least one of the four samples (Supplementary Table S2). In correlation analysis, NE values of the IFC and CFC ovaries were found to be highly correlated between repeats (*r* = 0.95 and 0.90, respectively; Supplementary Figure S1), indicating good reproducibility of the RNA sequencing results. A one-tailed *t*-test was used to identify differentially expressed miRNAs with a fold change >1.5 between IFC and CFC as well as a *p* value < 0.05 and an NE value > 5 in at least one of the samples. As a result, a total of 20 miRNAs were found to be differentially expressed between the two phenotypes (Table 2). Compared with the CFC ovary, 15 miRNAs were found to be down-regulated and 5 up-regulated in the IFC ovary (Table 2). Since the IFC and CFC ovaries were taken from the same area on the same ear, most of the detected miRNAs showed similar expression patterns; however, nevertheless, differences did exist.

**Table 2:** **Summary of the differentially expressed known miRNAs**.

**miR family**	**Members of the identified miRNAs**	**IFC (NE)**	**CFC (NE)**	***P*****-value**	**up/down**
miR156	ata-miR156b-3p_1ss6TC	16.21	7.67	0.038	up
	zma-miR156l-3p	22.34	3.71	0.000	up
miR159	zma-miR159a-3p	147.48	406.41	0.000	down
	zma-miR159c-3p_L-1R-1	928.11	471.01	0.000	up
miR160	osa-miR160a-5p_L+1	1.33	8.37	0.040	down
	zma-miR160f-5p_1ss21GA	89.55	144.19	0.010	down
miR164	osa-miR164d_R+1_1ss21TA	0.00	5.16	0.049	down
miR166	zma-miR166j-3p	507.86	838.61	0.000	down
miR168	zma-miR168a-5p	211.24	359.37	0.000	down
miR169	zma-miR169p-5p	31.30	117.39	0.000	down
miR171	zma-miR171d-5p	173.58	67.98	0.000	up
	osa-miR171i-5p	21.04	9.64	0.015	up
	gma-miR171m_1ss21AC	338.41	518.29	0.000	down
miR172	zma-miR172e	0.42	11.52	0.003	down
miR390	zma-miR390a-5p	200.96	391.99	0.000	down
miR393	zma-miR393b-5p_R-1	4.83	19.92	0.007	down
	zma-miR393c-3p_L-1	0.21	6.31	0.026	down
miR396	zma-miR396a-5p	96.75	165.23	0.002	down
miR399	zma-miR399c-5p	54.47	91.18	0.025	down
miR827	zma-miR827-5p_L+1	473.66	878.55	0.000	down

*IFC, incompletely fused carpels; CFC, completely fused carpels; NE, normalized expression*.

The miR_name is composed of the 1st known miR name in a cluster, a underscore, and a matching annotation. Such as:

L-/+ n, means the miRNA_seq (detected) is n base less or more than known rep_miRSeq in the left side, respectively;

R-/+ n means the miRNA_seq (detected) is n base less or more than known rep_miRSeq in the right side, respectively;

*1ss6TC13TA means 1 substitutin (ss), which are T->C at position 6 and T->A at position 13 from 5′ of miRNA*.

**Figure 3:**
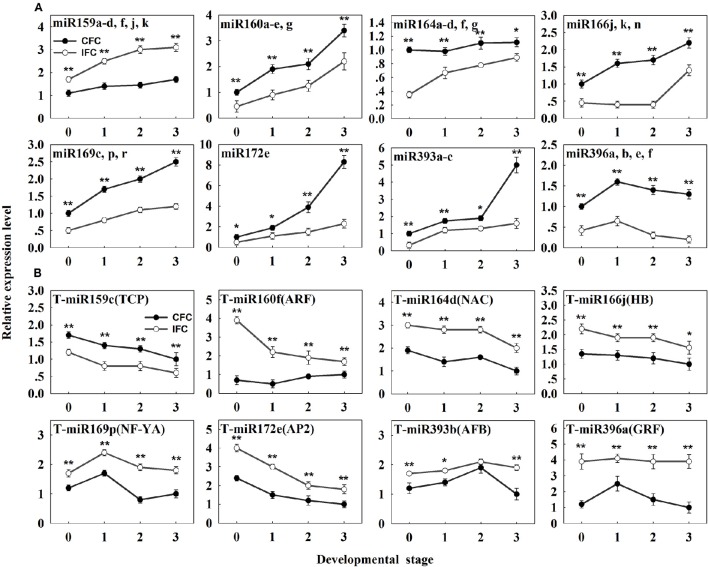
**qRT-PCR analysis of the identified differentially expressed known miRNAs and their targets in IFC and CFC ovaries. (A)** The copy number of miRNAs was normalized by comparison with maize U6. Relative expression levels of each miRNA were normalized by comparison with expression of the CFC ovaries at developmental stage “0,” which was set as 1. **(B)** The copy number of the corresponding target mRNAs was normalized by comparison with the maize gene *EF1a* (gene ID: GRMZM2G153541). Relative expression levels of each target were then normalized by comparison with expression of the CFC ovaries at developmental stage of “3,” which was set as 1. A primer pair spanning the cleavage site was used to quantify the expression of uncleaved target mRNA. Developmental stage “0” represents silking, when carpel fusion deficiency was initially observed. Developmental stages “1,” “2” and “3” represent the number of days after silking. Error bars represent the standard error. Samples used at developmental stage “0” were the same as those used for small RNA sequencing. Lowercase a-d, f, j, k, a-e and g after the miRNA name refer to highly homologous miRNAs. IFC, incompletely fused carpels; CFC, completely fused carpels; ^*^P < 0.05; ^**^P < 0.01.

To determine detailed expression patterns of these miRNAs in the IFC and CFC ovaries, real-time qRT-PCR was performed. Eight differentially expressed known miRNAs were selected for validation. Overall, the results corresponded to the deep sequencing results (Figure 3A), indicating reliability of the miRNA expression levels determined by high-throughput sequencing. In addition, we surveyed expression of these miRNAs in the IFC and CFC ovaries after the initial observation of carpel fusion deficiency, prior to pollination. Changes in expression levels of the selected miRNAs exhibited a consistent tendency with differential expression during IFC and CFC development. It should be noted that miR396 expression increased on the day of silking and thereafter decreased (Figure 3A).

### Target prediction and identification of miRNAs by degradome sequencing

To identify miRNA targets, two cleaved miRNA target libraries (degradomes) were generated for the IFC and CFC ovaries, respectively. After high-throughput sequencing, 16.9 and 16.7 million raw reads representing the 5′ ends of uncapped, poly-adenylated RNAs were obtained from the IFC and CFC libraries, respectively. After initial processing, 75.81% (75.88 and 75.73% for the IFC and CFC ovaries, respectively) of the short sequencing reads were mapped to the maize transcriptome, suggesting that some of the filtered reads mapped to unannotated genes (Supplementary Table S3).

**Figure 4:**
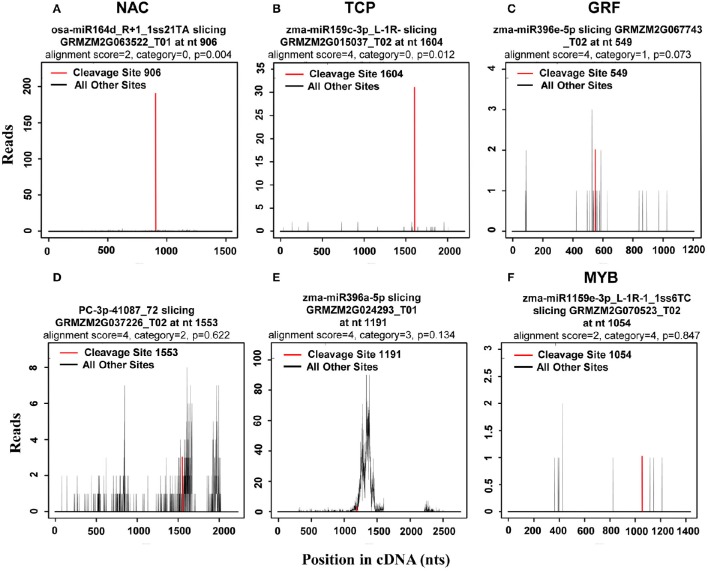
**Representative target plots (t-plot) depicting categories of the cleaved mRNAs confirmed by degradome sequencing, reflecting the reliability of the miRNA targets**. As previously reported (Karlova et al., 2013), cleavage products can be categorized into three classes: Class I t-plots, category 0 and 1 peaks with a *P* ≤ 0.05; Class II, category 0 and 1 peaks with a *P* > 0.05, and category 2 peaks; and Class III, category 3 and 4 peaks. Representative examples of **(A,B)** target genes belonging to Class I, **(C,D)** target genes belonging to Class II, and **(E,F)** target genes belonging to Class III are shown.

In plants, miRNAs have been shown to bind with almost complete complementarity to their mRNA targets, with miRNA-mediated cleavage occurring precisely between the 10th and 11th nucleotide from the 5′ end of the miRNA in the complementary region of the target mRNA. In the CleaveLand pipeline program (Addo-Quaye et al., 2009), sequenced tags were first mapped to cDNA then the number of tags with a 5′ nucleotide corresponding to each position in the mRNA sequence counted, as depicted by a t (target)-plot. That is, cleaved transcripts have distinct t-plot peaks at the predicted cleavage site in the degradome sequence tags. As previously reported (Karlova et al., 2013), the cleavage products can be categorized into three classes: Class I t-plots, category 0 and 1 peaks with a *P*-value ≤ 0.05; Class II, category 0 and 1 peaks with a *P*-value > 0.05, and all category 2 peaks regardless of their *P*-value; and Class III, all category 3 and 4 peaks regardless of their *P*-value. Thus, Class I includes the most credible miRNA target genes among the three classes. Representative examples of t-plots of cleavage transcripts belonging to the different classes are shown in Figure 4.

**Figure 5:**
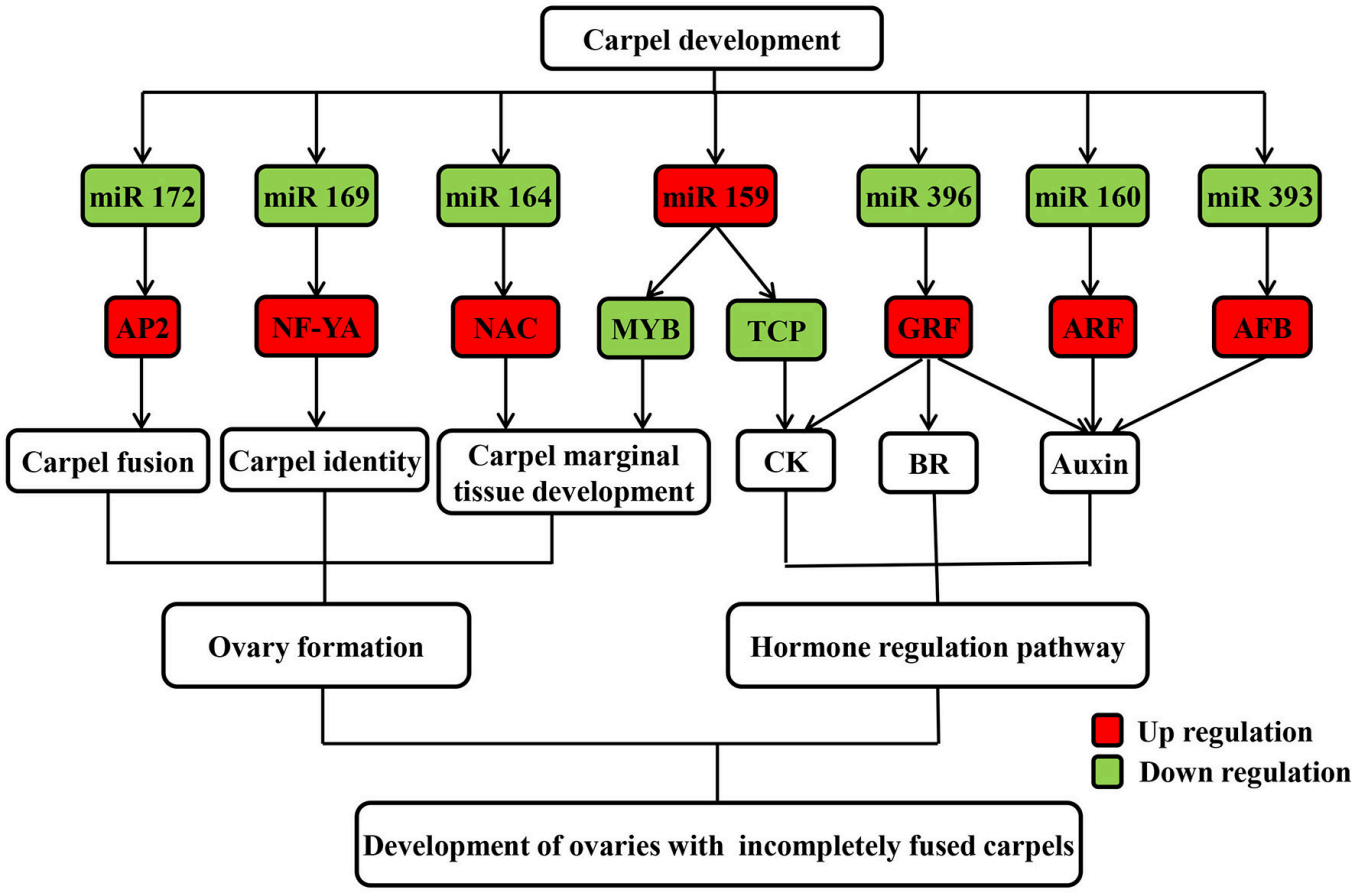
**Possible regulatory mechanism of differentially expressed known miRNAs and their targets involved in carpel fusion development in maize ovaries**.

As the results, a total of 113 (471 transcripts) target genes were predicted for 73 known miRNAs, 46 (181 transcripts) of which were categorized as Class I (Supplementary Table S4). Of 20 differentially expressed known miRNAs, degradome predication and analysis revealed that only 12 had target genes. Moreover, 20 (39 transcripts) target genes cleaved by nine differentially expressed known miRNAs were classified as Class I from a total of 40 (76 transcripts) identified target genes of all differentially expressed known miRNAs (Table 3; Supplementary Table S5). Sixty percent of these target genes were members of different families of transcription factors; namely, TCP, MYB, ARF, NAC, NF-YA, AP2, GRF, GRAS, and HD-ZIP (Table 3), while one gene, ARF, was found to be targeted by miR160 as verified by RNA ligase-mediated 5′-RACE (Supplementary Figure S2). Some of the differentially expressed known miRNAs targeted multiple gene loci; for example miR159 (Figure 5). Expression of eight miRNA target genes of differentially expressed known and novel miRNAs was subsequently detected using qRT-PCR analysis. As expected, expression of all target genes was negatively correlated with expression levels of the corresponding miRNA in both the IFC and CFC ovaries (Figures 3A,B, 5), This finding is also in accordance with the gene cleavage function of the miRNAs.

**Table 3:** **Identified target transcripts of differentially expressed known miRNAs by degradome analysis of IFC and CFC ovaries**.

**Target mRNA**	**Annotation^b^**	**TF family^c^**	**Cleavage position**	**Peak category**		**TPB**	
				**IFC**	**CFC**	**IFC**	**CFC**
**CLASS I**^a^							
**miR159**							
GRMZM2G089361_T01	TCP family transcription factor	TCP	10	0	0	473	1199
3030GRMZM2G020805_T01	TCP family transcription factor	TCP	10	0	0	2542	2458
GRMZM2G028054_T01, T02, T03	myb domain protein	MYB	10	0	0	709	580
GRMZM2G015037_T01, T02	TCP family transcription factor	TCP	10	0	0	473	929
AC217264.3_FGT005	myb domain protein	MYB	10	0	0	650	240
**miR160**							
GRMZM2G159399_T01	auxin response factor	ARF	10	0	0	52379	66723
GRMZM2G005284_T01	auxin response factor	ARF	10	1	0	177	120
**miR164**							
GRMZM2G063522_T01	NAC domain containing protein	NAC	10	0	0	9932	12170
**miR166**							
GRMZM2G038198_T01	Homeobox-leucine zipper protein		10	0	0	5819	7554
AC187157.4_FGT005	Homeobox-leucine zipper protein		10	0	0	709	899
**miR169**							
GRMZM2G040349_T01, T02	nuclear factor Y	NF-YA	10	0	0	207	150
GRMZM2G000686_T01, T02, T03, T04, T05, T06, T08	nuclear factor Y	NF-YA	10	0	0	177	187
**miR172**							
GRMZM2G076602_T01	DNA-binding protein	AP2	10	0	0	1064	480
**miR393**							
GRMZM2G137451_T01,T02	auxin signaling F-box / AFB		10	0	0	276	520
**miR396**							
GRMZM2G033612_T02	growth-regulating factor	GRF	11	0	0	10819	17865
GRMZM2G018414_T01, T02	growth-regulating factor	GRF	11	0	0	532	1529
GRMZM2G099862_T01, T02, T03, T04	growth-regulating factor	GRF	11	0	0	665	689
GRMZM2G041223_T01	growth-regulating factor	GRF	11	0	0	335	560
GRMZM2G034876_T01, T02, T03	growth-regulating factor	GRF	11	0	0	355	380
GRMZM2G129147_T01, T02	growth-regulating factor	GRF	11	0	0	2256	2987
**CLASS II**							
**miR159**							
GRMZM2G004090_T01	myb domain protein	MYB	10	2	0	118	210
GRMZM2G028054_T01, T02, T03	myb domain protein	MYB	10	2	2	117	240
**miR160**							
GRMZM2G159399_T01	auxin response factor	ARF	10	2	2	946	1079
**miR166**							
GRMZM2G552083_T01	Homeobox-leucine zipper protein		10	2	0	25	188
GRMZM2G123644_T01	agenet-containing protein		10	2	2	25	188
GRMZM2G336718_T01	agenet-containing protein		10	2	2	25	188
**miR171**							
GRMZM2G051785_T01	GRAS transcription factor	GRAS	10	0	2	0	180
GRMZM2G110579_T01	GRAS transcription factor	GRAS	10	2	2	635	555
GRMZM2G037792_T01	GRAS transcription factor	GRAS	10	2	2	315	360
GRMZM5G825321_T01, T02	GRAS transcription factor	GRAS	10	2	2	296	360
**miR393**							
GRMZM5G848945_T02	auxin signaling F-box / AFB		10	2	2	276	520
**miR396**							
GRMZM2G067743_T01, T03	growth-regulating factor	GRF	11	2	0	39	160
GRMZM2G067743_T02	growth-regulating factor	GRF	11	1	0	39	160
**Class III**							
**miR159**							
GRMZM2G113073_T01	P21-Rho-binding domain		10	2	4	177	60
GRMZM2G127720_T01	—		10	4	0	59	0
AC217264.3_FGT005	myb domain protein	MYB	10	0	4	0	60
GRMZM2G070523_T01, T02, T03	myb domain protein	MYB	10	4	0	20	0
**miR166**							
GRMZM2G003509_T01, T02	Homeobox-leucine zipper protein	HD-ZIP	10	3	2	25	231
**miR169**							
GRMZM2G037630_T01	nuclear factor Y, subunit A6	NF-YA	10	4	0	59	120
**miR171**							
GRMZM2G000039_T01	SIT4 phosphatase-associated protein		10	3	4	20	40
**miR172**							
GRMZM5G856084_T01	DNAJ heat shock family protein		10	4	0	59	0
**miR390**							
GRMZM2G121820_T01	Leucine-rich repeat family protein		10	0	4	0	20
GRMZM5G815009_T01, T02	Leucine-rich repeat family protein		10	0	4	0	20
**miR396**							
GRMZM2G024293_T01, T03	P-loop nucleoside triphosphate hydrolases		10	3	3	59	90

*A comprehensive list could be found in Supplementary Table S5*.

a *Classification of the three different classes was made according to Karlova et al. (2013)*.

b *Putative functions were derived from Ensembl Plants (plants.ensembl.org, release 28)*.

c *TF family members were classified according to the Plant Transcription Factor Database v3.0 (Jin et al., 2013)*.

*IFC, incompletely fused carpels; CFC, completely fused carpels; TPB, tags per billion*.

### Identification of potential novel miRNAs

To identify novel miRNAs, sequencing reads that did not match any of the known miRNAs were further analyzed with reference to the criteria for annotation of plant miRNAs (Meyers et al., 2008). A total of 43 miRNA candidates, present in at least one of the four libraries, were revealed and designated predicated candidates (PC)-5P/3P. In addition, 10 miRNA new isoforms in known miRNA loci were also identified (Supplementary Table S6). Secondary structures of eight of the selected novel miRNAs are presented in Supplementary Figure S2. Both miRNAs of predicated candidates and new isoforms were defined as novel miRNAs. Of these, a total of 10 were differentially expressed between the IFC and CFC ovaries (Supplementary Table S7).

**Figure 6:**
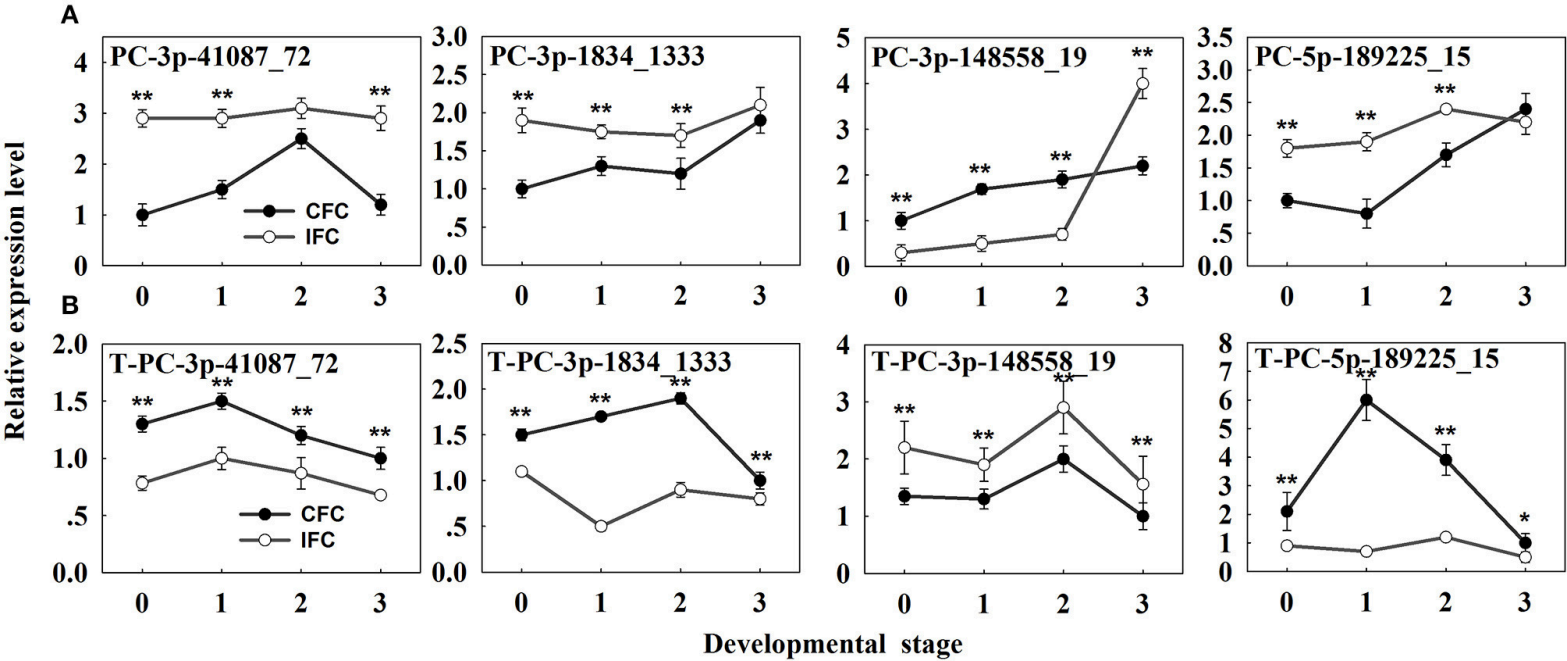
**qRT-PCR analysis of the identified differentially expressed novel miRNAs and their targets in IFC and CFC ovaries. (A)** The copy number of the miRNAs was normalized by comparison with maize U6. Relative expression levels of each miRNA were normalized by comparison with expression of the CFC ovaries at developmental stage of “0,” which was set as 1. **(B)** The copy number of corresponding target mRNAs was normalized by comparison with the maize gene *EF1a* (gene ID: GRMZM2G153541). Relative expression levels of each target were then normalized by comparison with expression of the CFC ovaries at developmental stage “3,” which was set as 1. A primer pair spanning the cleavage site was used to quantify the expression of uncleaved target mRNAs. Developmental stage “0” represents silking, when carpel fusion deficiency was initially observed. Developmental stages “1,” “2,” and “3” represent the number of days after silking. Error bars represent the standard error. Samples used at developmental stage of “0” were the same as those used for small RNA sequencing. IFC, incompletely fused carpels; CFC, completely fused carpels; ^*^P < 0.05; ^**^P < 0.01.

Potential targets of the novel miRNAs were also predicted via degradome sequencing. A total of 11 target genes (14 transcripts) of 9 novel miRNAs were identified, all belonging to Classes II and III (Supplementary Table S8). However, only seven targets of four differentially expressed novel miRNAs were revealed (Supplementary Table S9). These results clearly differ from the identified targets of known miRNAs (Table 3; Supplementary Table S9; Figure 4). The four differentially expressed novel miRNAs and their target genes were subsequently selected for qRT-PCR analysis (Figure 6A), revealing a negative correlation between all miRNAs and their corresponding target genes except for PC-5p-189225_15 (Figure 6B). This exception was possibly due to its low expression level as observed in the sRNA sequencing results (Supplementary Table S7).

### Dynamic changes in phytohormones during IFC and CFC ovary development

**Figure 7:**
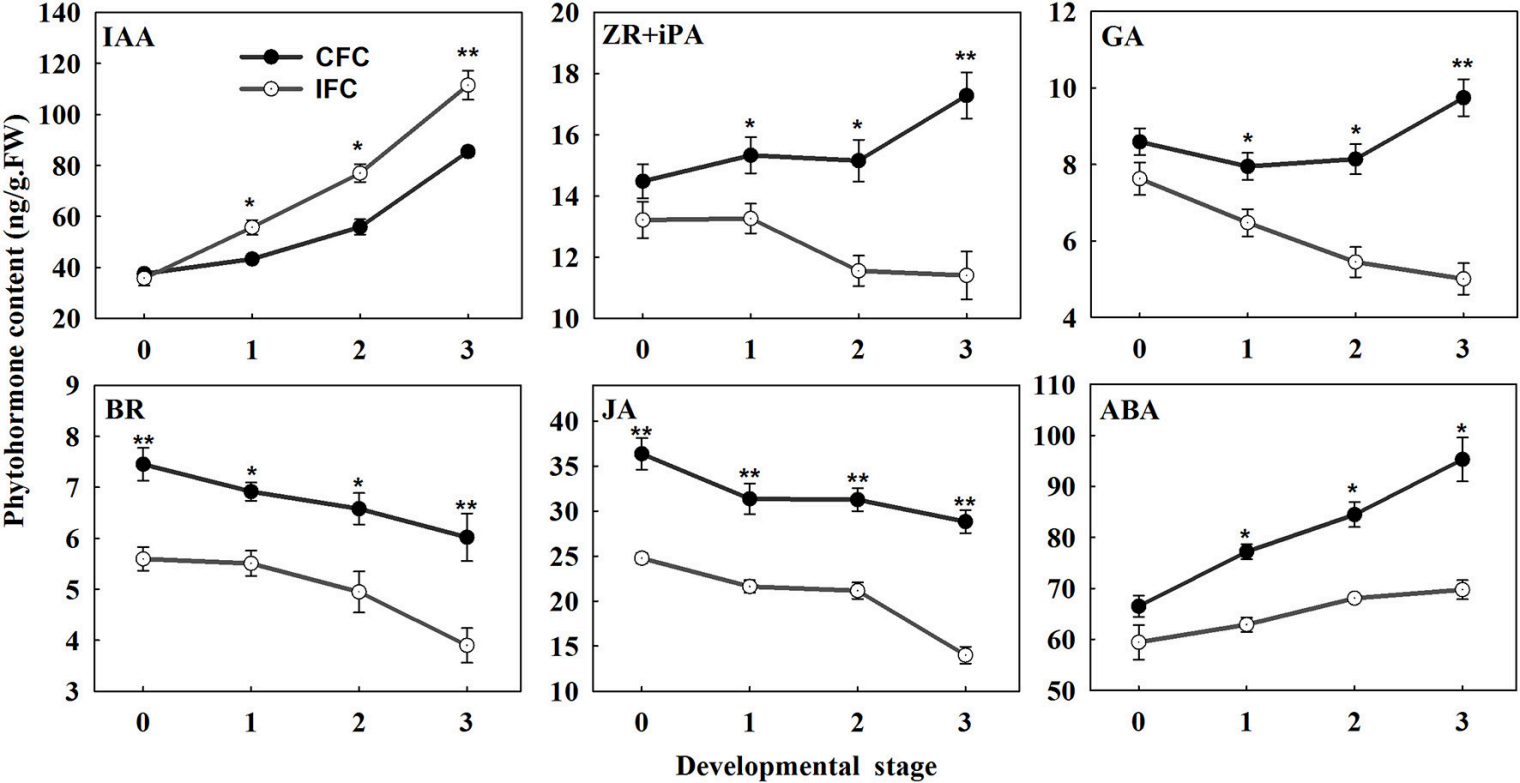
**Phytohormone contents at different developmental stages during ovary development in IFC and CFC ovaries**. Developmental stage “0” represents silking, when carpel fusion deficiency was initially observed. Developmental stages “1,” “2,” and “3” represent the number of days after silking. Error bars represent the standard error. Samples used at developmental stage of “0” were the same as those used for small RNA sequencing. IFC, incompletely fused carpels; CFC, completely fused carpels; ^*^P < 0.05; ^**^P < 0.01.

To examine the changes in endogenous phytohormone accumulation during deficient carpel fusion, we compared the contents of various endogenous phytohormones in IFC and CFC ovaries at silking (when the deficiency could be observed in serial observations) and 1, 2. and 3 days after silking, respectively. In general, the endogenous IAA content of the IFC ovaries was significantly higher than that of the CFC ovaries at each developmental stage except the initial observation at silking. In contrast, the contents of all other endogenous phytohormones, including zeatin riboside and isopentenyl adenine (ZR + iPA), GA, brassinosteroids (BR), jasmonic acid (JA) and abscisic acid (ABA), were lower in the IFC ovaries. The pattern of IAA accumulation was also similar between the IFC and CFC ovaries, both showing a gradual increase; however, the changes in ZR + iPA and GA contents showed different patterns. In the CFC ovaries, ZR + iPA and GA increased with ovary growth, whereas a reverse trend was observed in the IFC ovaries. Changes in endogenous BR, JA and ABA contents showed consistent tendencies, with a decrease in BR and JA and an increase in ABA in both the IFC and CFC ovaries (Figure 7).

### Correlation analysis of phytohormone contents, miRNAs and their targets

Target gene expression was examined using qRT-PCR during ovary formation, prior to pollination. We then focused our attention primarily on miRNAs and their target genes in relation to phytohormone synthesis and metabolism, since they were deemed most likely to play important roles in carpel fusion development. To further investigate the potential relationship between phytohormone signals and the expression of miRNAs and their target genes during IFC ovary development, we selected eight differentially expressed known miRNAs and their targets for correlation analysis. As a result, expression patterns of all 8 miRNAs and 8 corresponding targets were found to be significantly correlated with the phytohormone signals (Table 4). Furthermore, these miRNAs and their target genes were subsequently divided into two groups. In the first, the miRNAs and their corresponding targets showed opposite correlation relationships with the phytohormone signals. That is, they all (miR159 and its target with IAA; miR166, miR169, miR393, miR396, and their targets with ZR + iPA; miR159, miR396 and their targets with GA; miR396 and their targets with BR; miR159, miR396 and their targets with JA; miR160, miR164, miR166, miR169, miR172, and miR396 and their targets with ABA) showed a significant positive and/or negative correlation with the phytohormone levels. In contrast, the second group included only those miRNAs or targets that were significantly correlated with the phytohormone signals. For example, miR160 was significantly correlated with IAA, but its target gene was not. Taking into account both the miRNAs and their targets, it should be noted that either the selected miRNA and / or its target had a significant correlation with at least one phytohormone signal (Table 4).

**Table 4:** **Correlation analysis of expression of eight differentially expressed known miRNAs and their targets with phytohormone levels (*n* = 8)**.

**miRNA/Target^a^**	**IAA^b^**	**(ZR+iPA)^b^**	**GA^b^**	**BR^b^**	**JA^b^**	**ABA^b^**
miR159a-d, f, j, k	0.73^*^	−0.77^*^	−0.88^**^	−0.93^**^	−0.94^**^	−0.29
miR160a-e, g	0.61^*^	0.55	0.34	−0.05	0.04	0.93^**^
miR164a-d, f, g	0.28	0.55	0.39	0.39	0.44	0.79^**^
miR166j, k, n	0.31	0.72^*^	0.57	0.26	0.34	0.91^**^
miR169c, p, r	0.32	0.74^*^	0.55	0.26	0.34	0.99^**^
miR172e	0.45	0.70^*^	0.55	0.06	0.16	0.93^**^
miR393a-c	0.47	0.68^*^	0.52	0.06	0.14	0.91^**^
miR396a, b, e, f	−0.38	0.88^**^	0.77^*^	0.80^**^	0.79^**^	0.68^*^
T-miR159c (TCP) GRMZM2G089361_T01	−0.79^**^	0.54	0.69^*^	0.94^**^	0.94^**^	0.07
T-miR160f (ARF) GRMZM2G005284_T01	−0.11	−0.51	−0.36	−0.51	−0.52	−0.63^*^
T-miR164d (NAC) GRMZM2G063522_T01	−0.20	−0.77^*^	−0.62^*^	−0.43	−0.49	−0.88^**^
T-miR166j (HB) AC187157.4_FGT005	−0.18	−0.76^*^	−0.61^*^	−0.48	−0.53	−0.89^**^
T-miR169p (NF-YA) GRMZM2G040349_T01,T02	0.06	−0.66^*^	−0.71^*^	−0.52	−0.64^*^	−0.70^*^
T-miR172e (AP2) GRMZM2G076602_T01	−0.48	−0.46	−0.24	−0.14	−0.20	−0.87^**^
T-miR393b (AFB) GRMZM2G137451_T01, T02	0.22	−0.80^**^	−0.83^**^	−0.61	−0.64^*^	−0.46
T-miR396a (GRF) GRMZM2G033612_T02	0.18	−0.84^**^	−0.85^**^	−0.74^*^	−0.82^**^	−0.73^*^

a *Putative functions were derived from Ensembl Plants (plants.ensembl.org, release 28) and TF family members were classified according to the Plant Transcription Factor Database v3.0 (Jin et al., 2013)*.

b *IAA, (ZR+iPA), GA, BR, JA, and ABA represent the tendencies of relative coefficients, respectively, between endogenous phytohormone and expression patterns of miRNAs and their targets*.

* *P < 0.05*;

** *P < 0.01. Lowercase a-d, f, j, k, a-e and g after the miRNA name refer to highly homologous miRNAs*.

## Discussion

With the development of sequencing technologies, sRNA sequencing has become an efficient and economical technique for estimating expression profiles of miRNA genes. Recent studies have reported the role of miRNAs in determining gene expression during maize ear and kernel development (Liu et al., 2014; Xin et al., 2015). However, few studies have examined miRNA expression during ovary growth, in particular, during carpel fusion. In this study, we conducted deep sequencing of sRNAs in IFC and CFC ovaries at the moment of initial carpel fusion deficiency. As a result, 20 differentially expressed known miRNAs were identified, all of which cleaved target genes related mainly to TFs. Furthermore, a large number of these differentially expressed miRNAs and their targets showed a significant correlation with endogenous hormones during ovary development. These findings suggest that miRNAs, especially those differentially expressed between IFC and CFC ovaries, may play crucial roles in regulating ovary development.

### Analysis of the identified miRNAs and their target genes

miRNAs, a class of sRNAs, were previously shown to play an important role in controlling gene expression via cleavage to a target gene(s), many of which are members of TF families (Bartel, 2004; Kidner and Martienssen, 2005). In this study, we obtained an average of 2.7 and 3.6 million unique valid reads for 18–26 nt sequences in the IFC and CFC libraries, respectively, substantially increasing the available data on maize ovary sRNAs. Analysis of these unique sRNAs by mapping to miRBase revealed that most of the identified miRNAs were previously unannotated, suggesting that many more remain to be explored. In comparison, a total of 30 differentially expressed miRNAs were revealed, of which 20 were known and 10 were novel. These findings suggest that these known miRNAs may play a role in the completion of carpel fusion during ovary development, as further suggested by their annotations in degradome sequencing (Table 3). However, some of these newly identified miRNAs probably had a specific role in carpel fusion or had no action, as implied by the degradome analysis (Supplementary Table S9), this might due to the lower expression of these novel miRNAs and/or the function of these novel miRNAs' targets on ovary development were not fully studied.

As mentioned above, some of the identified novel miRNAs showed relatively low expression, consistent with previous observations suggesting that novel miRNAs are often expressed at lower levels than conserved miRNAs (Pantaleo et al., 2010; Liu et al., 2014). miRNAs showing low levels of expression are generally thought to have limited biological function, and therefore, their effects on the target genes would, in most cases, be weak or negligible (Hackenberg et al., 2015). However, despite this, we cannot rule out the possibility that these miRNAs participate in carpel fusion development in maize ovaries.

Degradome sequencing is a relatively powerful tool for identifying potential targets, and was used here to identify the functions of candidate miRNAs using samples from IFC and CFC ovaries. As the result, a total of 181 transcripts of 46 target genes were identified as Class I miRNAs (Supplementary Table S4). The remaining transcripts all belonged to Classes II and III, the latter of which are considered low confidence miRNA-target pairs (Karlova et al., 2013; Baksa et al., 2015). These findings were possibly due to the complex processes of transcriptional regulation, or the limitations of target identification with degradome sequencing.

### TFs targeted by differentially-expressed known miRNAs might be involved in regulation of carpel fusion

Most gynoecia require complete carpel fusion to ensure ovary formation. miRNAs are important regulators of carpel fusion during gynoecium development (Sieber et al., 2007; Larue et al., 2009). For example, a previous study showed that maize *ts4*, which encodes miR172, plays a key role in carpel fusion in pistils of tassel flowers (Chuck et al., 2007). Moreover, the TF *AP2*, one identified target of miR172, was found to be involved in carpel fusion during *Arabidopsis* gynoecium development (Ripoll et al., 2011). It was also revealed that miR156 controls the initial steps of fleshy fruit development in tomato, playing an important role in ovary and fruit development (Ferreira et al., 2014). Moreover, two targets of miR156 were found to positively regulate miR172 expression by binding their sequence to the regulatory region of miR172 (Wu et al., 2009). In our study, expression of miR172 was down regulated, while that of miR156 was up regulated in IFC compared to CFC ovaries (Table 2), suggesting a similar interaction between miR172 and miR156 may exist in maize ovary as well. Overall, the degradome and qRT-PCR analyses suggest that the differentially expressed miR172 might also regulate carpel fusion during maize ovary development by targeting *AP2*.

Carpel fusion requires coordination among various functional genes. A large proportion of the genes known to be involved in normal carpel fusion are TF genes, two of which, MYB and TCP, are known to play a key role in regulation of carpel fusion development in *Arabidopsis* (Shin et al., 2002; Koyama et al., 2007), were found to be targeted by miR159 (Table 3). Moreover, mRNA expression of both TFs was found to show an opposite trend compared to miR159 during ovary development in both IFC and CFC ovaries (Figures 3, 5), suggesting a consistent regulatory role of miR159 during ovary development in maize. miR164 and its target gene, NAC, have been described in relation to carpel marginal tissue development in *Arabidopsis* (Sieber et al., 2007). Furthermore, CUC2, a member of the NAC family, is known to be a key regulator of carpel fusion development in *Arabidopsis* (Nahar et al., 2012). In addition, miR169 and its targets, members of the NF-YA family of TFs, were previously found to exert homeotic control over the carpel identity gene *AG* in *Petunia hybrida* and *Antirrhinum majus* (Cartolano et al., 2007). Overexpression of miR169 was also found to cause changes in fruit shape and size in tomato (Teotia et al., 2015). In this study, AP2, MYB and TCP, and other TF family members including NAC, ARF, GRF, and NF-YA, were identified as Class I target genes of the differentially expressed known miRNAs (Table 3). Taken together, these findings suggest that these miRNAs may play a vital role in controlling carpel fusion development.

### Differentially expressed miRNAs may control ovary development through regulation of phytohormone homeostasis

During formation and growth of the maize pistil, a period of 3 days after silking is sufficient for pollination of the maximum number of whole ear kernels (Culy et al., 1992). Thus, at this developmental stage, the pistil undergoes appropriate ovary development, with sufficient silk vitality and silk length, and develops sufficiently for fertilization (Culy et al., 1992). Therefore, understanding phytohormone levels at this stage as well as expression levels of miRNAs and their targets is important, as is determining their interaction and regulation in IFC and CFC ovary development.

In cereals, carpel fusion and gynoecium development is reportedly regulated by plant hormones (Barazesh and Mcsteen, 2008; Larsson et al., 2013; Reyes-Olalde et al., 2013; Marsch-Martínez and de Folter, 2016). Accumulating evidence suggests that miRNAs affect plant hormone signaling and hormone synthesis, In addition, sensing and transport of these hormones relies on the activity of proteins, with most genes involved in this regulatory network regulated by miRNAs (Curaba et al., 2014; Marsch-Martínez and de Folter, 2016).

Auxin, which stimulates cell differentiation, is extremely important to plant development, particularly gynoecium development (Hawkins and Liu, 2014; Sehra and Franks, 2015). Experimental evidence suggests that auxin also plays a crucial role in carpel marginal tissue development in *Arabidopsis* (Reyes-Olalde et al., 2013). In rice, ARF6 controls inflorescence development through coordinated activation of auxin biosynthesis and auxin response factors (Gao et al., 2015). ARF8 and ARF6, important signaling components in the auxin signaling pathway, are closely related TFs both targeted by miR167, and involved in carpel maturation and development in *Arabidopsis* (Nagpal et al., 2005). Our results showed no difference in IAA content when carpel fusion deficiency was first observed; however, soon after, the content became significantly higher in IFC than CFC ovaries (Figure 7). This is consistent with previous studies suggesting that a high concentration of auxin is essential for carpel fusion (Larsson et al., 2013; Sehra and Franks, 2015). Gene expression of auxin response factor (ARF), a target gene of miR160, was also higher in IFC ovaries (Table 2; Figure 3), indicating a major role in auxin homeostasis and carpel fusion. This is consistent with a previous study showing that mutation in the *ARF* gene can lead to alterations in carpel and gynoecium development, and patterning in *Arabidopsis* (Nagpal et al., 2005; Crawford and Yanofsky, 2011).

Cytokinins (CKs) are a structurally diverse species of N^6^-substituted purine derivatives that stimulate mitotic divisions, and also known to participate in carpel marginal tissue development (Reyes-Olalde et al., 2013; Marsch-Martínez and de Folter, 2016). *OsGRF4*, a target of miR396, reportedly regulates a CK dehydrogenase precursor gene and controls rice grain shape (Sun et al., 2016), while TCP14 and TCP15 TFs, which mediate CK responses, were found to cause excessive proliferation of the boundaries of the *Arabidopsis* gynoecium replum (Takei et al., 2001). Isopentenyl adenine (iPA) and zeatin riboside (ZR) are the major CK species in maize (Takei et al., 2001). Our data showed lower iPA and ZR contents in IFC ovaries except at the initial stage of incomplete carpel fusion (Figure 7), which is possibly related to the gradual increase in morphological differences between the IFC and CFC ovaries. Our data also revealed that expression of TCP, the target gene of miR159, was higher in IFC than CFC ovaries (Table 2; Figure 3), suggesting a regulatory function of miR159 in carpel fusion development.

Gibberellin (GA) is thought to play diverse roles in plant growth and development, including flowering time, with overexpression of miR156 reducing GA responses during flowering (Yu et al., 2012). The *GA3ox1* gibberellin biosynthesis gene is a direct target of INDEHISCENT (IND) TF; however, *ind* mutant plants, which have low GA levels, show abnormal carpel valve margin development in the *Arabidopsis* gynoecium (Arnaud et al., 2010). The role of brassinosteroids (BR) in gynoecia development was also recently examined. For example, *OsGRF4*, a target of miR396, was found to positively regulate BR content through direct interaction with *GSK2*, the central negative regulator of brassinosteroid signaling in rice (Che et al., 2015). The enzyme CYP85A2 is also known to participate in brassinolide biosynthesis, with the double mutant *seu cyp85A2* causing carpel fusion defects in the gynoecial apex of *Arabidopsis* (Nole-Wilson et al., 2010). Furthermore, decreased production of the plant hormone JA reportedly accounts for a subset of *arf6 arf8* mutant phenotypes, including developmental defects in carpels, (Nagpal et al., 2005). Moreover, in *Arabidopsis*, it is reported that *ARF6* and *ARF8* are directly regulated by miR167 at the post-transcription level (Wu et al., 2006). miR159 also targets genes encoding the MYB TF, which is involved in regulation of ABA (Reyes and Chua, 2007). ABA was also shown to have a positive effect on flowering initiation and a negative effect on flower development (Wang et al., 2002). Therefore, these miRNAs and their targets as well as phytohormones all together may contribute to a balanced manner during ovary formation and development, with interruption of any one of them might result in an abnormal ovary development.

In conclusion, in this study, we performed genome-wide identification of known and novel miRNAs and experimentally predicated their targets through degradome analysis. Furthermore, the changes in endogenous phytohormone contents between IFC and CFC ovaries were also examined, and the correlation with miRNAs and their targets determined. The presence of differentially expressed miRNAs during transcription regulation and phytohormone signaling processes in IFC and CFC ovaries suggests the significant roles of miRNAs in carpel fusion development. This study provides valuable information on carpel fusion development that will be beneficial in breeding programs aimed at enhanced seed vigor and quality in maize. Further genetic studies are now needed to determine the nature of these regulatory interactions.

## Materials and methods

### Plant materials

*Zea mays* (maize) inbred line Yu-A474 was planted in the farm of Henan Agricultural University, Zhengzhou city (Henan Province, China), under non-stress conditions during June-October 2014. This inbred line is a female of the maize hybrid Yudan 603, some ovaries of which show various phenotypes of incompletely fused carpels. Before silk emergence, ears were bagged to prevent pollination. To determine the timing of initial emergence of the IFC phenotype, we carried out continuous and repeated microscopic observations from the stage of floret primordium differentiation to floret organ differentiation during ear development in August 2013 (Supplementary Figure S3). Ovary samples were collected at silking, the time at which carpel wall fusion deficiency was initially observed. Briefly, ovaries were manually collected at the base using forceps after removal of the glumes, lemma and palea. To eliminate inconsistencies from sampling different parts of the ear, IFC ovaries were sampled close to the CFC ovaries, in the middle part of the same ear. All collected ovaries were immediately frozen in liquid nitrogen and stored at −80°C for RNA isolation. Two replicates from two different ear rows were collected, respectively, for the two ovary phenotypes. In total, four samples, including two biological replicates from CFC and IFC, respectively, were collected and prepared for total RNA extraction.

To confirm expression patterns of the 12 differentially expressed miRNAs and their target genes, IFC and CFC ovaries were collected on the day of silking then 1, 2, and 3 days after silking, prior to pollination. This ensured that the period of ovary formation and growth was covered as well as meeting the conditions of fertilization.

### RNA isolation, small RNA library construction and sequencing

Total RNA for sRNA sequencing, qRT-PCR and degradome analyses was extracted from IFC and CFC ovaries using Trizol reagent (Invitrogen) according to the manufacturer's instructions. Quality and purity of the total RNA was analyzed using Bioanalyzer 2100 (Agilent) and an RNA 6000 Nano LabChip Kit (Agilent). From each sample, 1 μg of total RNA was ligated to RNA-DNA chimeric oligonucleotide adaptors then converted to cDNA by RT-PCR. The resulting cDNA was amplified by PCR and gel-purified to produce sequencing libraries. Finally, sRNA sequencing (single-end, 50 bp) was performed on an Illumina Hiseq2500 (Illumina) platform according to the manufacturer's recommended protocol.

### Identification of known miRNAs

To identify known miRNAs, the following were performed. Raw reads were subjected to the Illumina pipeline filter (Solexa 0.3) then the dataset processed using ACGT101-v4.2-miR (LC Sciences, Houston, Texas, USA) to remove adapter sequences, junk reads, reads less than 15 nt, common RNA families (rRNA, tRNA, snRNA, and snoRNA), repeats and sRNA reads assigned to exon regions. The remaining small RNAs were classified by alignment to mRNA, RFam and Repbase databases then filtered.

The last remaining unique sequences, which were 18–25 nt in length, were mapped to maize precursors in miRBase 21.0 (ftp://mirbase.org/pub/mirbase/CURRENT/) using a BLAST search to identify known miRNAs and new isoforms. Length variation at the 3′ and 5′ ends and one mismatch inside the sequence were allowed in the alignment. Unique sequences mapped to mature maize miRNAs in hairpin arms were classified as known miRNAs. Unique sequences mapped to the other arm of known maize precursor hairpins, opposite the annotated mature miRNA-containing arm, or to different positions on the same arm were considered new isoforms. Remaining sequences were mapped using bowtie, an alignment tool in the proprietary pipeline script ACGT101-v4.2-miR (LC Sciences, Houston, Texas, USA), to precursors of other selected species (with the exclusion of maize) to identify known and new isoforms (one mismatch inside these sequences was allowed with a seed length of 16 nt). The identified precursors were further mapped to the maize genome using Megablast, another alignment tool in ACGT101-v4.2-miR (LC Sciences, Houston, Texas, USA), to determine their genomic locations. Only those showing a similarity rate of more than 90% were selected.

### Identification of potential novel miRNAs

After identification of known and new isoforms, remaining sequencing reads that did not match any known miRNA precursors were subjected to “ACGT101-v4.2-miR” to further determine novel miRNAs. Criteria were mainly those of Meyers and Lee (Meyers et al., 2008; Peng et al., 2013). Parameters for detailed identification of secondary structures were as follows: (1) number of nucleotides in one bulge in the stem region <13; (2) number of base pairs in the stem region of the predicted hairpin >15; (3) cutoff of free energy during the formation of secondary structures < −15 kcal/mol; (4) length of the hairpin (up and down stems + terminal loop) >49; (5) length of the hairpin loop <351 nt; (6) number of nucleotides in one bulge in a mature region <5; (7) number of biased errors in one bulge in a mature region <3; (8) number of biased bulges in a mature region <3; (9) number of mismatch errors in a mature region <5; (10) number of base pairs in a mature region of the predicted hairpin >11; and (11) percentage of mature base numbers in the stem >80%. Furthermore, unmapped sequences were BLASTed against the maize genome, and the mapped sequences containing hairpin RNA structures predicated from flank 120-nt sequences using mfold software (part of ACGT101-v4.2-miR).

### Analysis of differentially expressed miRNAs

To compare differentially expressed miRNAs between IFC and CFC, expression abundances were normalized to obtain the expression of transcripts per 1,000,000 using the following formulae:

Normalized expression (NE) = (Actual miRNA reads count/Total count of clean reads) × 1,000,000.

Before identification of differentially expressed miRNAs, we conducted reproducibility analysis of data from two replicate IFC and CFC ovaries using SCC analysis (Zhan et al., 2015). Log_2_-transformed NE values [log_2_ (NE + 1)] of the expressed miRNAs were used as input for the SCC analysis. Differential expression was analyzed using a *t*-test, based on normalized counts with a significance threshold set as a fold change of NE >1.5 and a *P*-value < 0.05 (Wang et al., 2012; Boke et al., 2014; Chang et al., 2015).

### Degradome library construction and bioinformatics analysis

Two degradome cDNA libraries were constructed from the same ovary samples used for sRNA analysis, mixing the two biological samples into one for IFC and CFC, respectively. Single-end sequencing (50 bp) was performed on an Illumina Hiseq2500 (Illumina) according to the method of German et al. (2008) with some modifications. Briefly, the extracted poly (A) RNA was ligated to a 5′ adapter containing a MmeI site at its 3′end. The ligated products were then used for cDNA production and amplified by PCR for 5 cycles. The PCR products were purified and digested with MmeI, and the resulting fragments ligated to a second double-stranded DNA oligonucleotide. The ligation products were further purified and amplified for another 10 PCR cycles, and the final product purified and subjected to high throughput sequencing.

The publicly available CleaveLand pipeline version 3.0.1 software package (Addo-Quaye et al., 2009) and Target Finder program (http://targetfinder.org/) (Kiełbasa et al., 2010) were used to detect potentially sliced targets of the known and novel miRNAs identified in sRNA sequencing. To account for inaccurate target cleavage or variations in miRNA 5′ ends, the pipeline was modified to recognize targets cleaved at the 9th, 11th, and 10th positons. All targets were classified as t-plot peaks according to 5 categories (0–4) based on the abundance of the resulting mRNA tags relative to the overall profile of the degradome reads matching the target (Addo-Quaye et al., 2008). Classification was as follows: peaks in categories 0–3, >1 read per peak; category 0, peaks representing a single maximum in a particular transcript; category 1, peaks equal to the maximum, with more than one maximum per transcript; category 2, peaks lower than the maximum but higher than the median of a transcript; and category 3, peaks with an equal or less than median number of reads. Category 4 peaks had only 1 read. The statistical significance of an observed peak-miRNA match was represented by a *P*-value < 0.05.

### Quantitative real-time RT-PCR (qRT-PCR)

Expression levels of the differentially expressed miRNAs and the predicated targets were validated by qRT-PCR analysis. Eight known miRNAs (miR159, miR160, miR164, miR166, miR169, miR172, miR393, and miR396), four newly identified miRNAs, and 12 target genes were selected for qRT-PCR validation. qRT-PCR was performed using the SYBR Green PCR Master Mix (Applied Biosystems) and an ABI PRISM_7900 Sequence Detection System (ABI, USA) following the manufacturer's instructions. To detect the level of non-cleaved mRNAs, all primers designed for the target genes spanned the miRNA cleavage site. Primer sequences of the miRNAs and their targets are listed in Supplementary Table S10. U6 RNA and *EF1a* (gene ID: GRMZM2G153541) were used as an internal reference for miRNA and their target genes, respectively. All reactions were performed in triplicate for technical and biological repetition of the IFC and CFC ovaries, respectively, and the generated real-time data analyzed using the comparative 2^−ΔΔCt^ method.

### RNA ligase-mediated 5′ race

To determine the cleavage sites of the target transcripts, we performed RNA ligase-mediated rapid-amplification of 5′ complementary DNA ends (5′-RLM-RACE) with a GeneRacer kit (Invitrogen, Carlsbad, CA, USA) according to the manufacturer's instructions with slight modifications. Briefly, total RNA from mixtures of IFC and CFC ovaries were ligated directly to a 5′ RACE RNA adapter (5′-GCTGATGGCGATGAATGAACACTGCGTTTGCTGGCTTTGATGAAA-3′) followed by reverse transcription with the GeneRacer (dT) primer. The reverse transcription product was used as template for PCR, with GeneRacer™ 5′ Primer (5′-GCTGATGGCGATGAATGAACACTG-3′), GeneRacer™ 5′ Nested Primer (5′-CGCGGATCCGAACACTGCGTTTGCTGGCTTTGATG-3′), and two gene-specific reverse primers used in each RACE (Supplementary Table S10). The RACE products were gel-purified, cloned to the pGEM-T Easy vector (Promega, Madison, WI, USA), and 10 independent clones from each reaction sequenced.

### Detection of phytohormones

Samples used for phytohormone measurement were the same as those used for qRT-PCR validation. Approximately 0.2 g of frozen samples were ground and homogenized in 2 ml of 80% methanol extraction medium (containing 40 mg l^−*l*^ butylated hydroxytoluene as an antioxidant). The extract was incubated at 4°C for 24 h then centrifuged at 4,000 revolutions per minute for 15 min at 4°C. The supernatant was passed through Chromosep C18 Sep-Pak cartridges (Waters Corp., Millford, MA, USA) then prewashed with 10 ml of 100% (v/v) methanol followed by 10 ml 80% (v/v) methanol. The eluate was dried under pure N_2_ and the extracts dissolved in 2.0 mL phosphate-buffer saline (PBS) (pH 7.5) containing 0.1% (v/v) Tween 20 and 0.1% (w/v) gelatin to examine free IAA, ZR, iPA, GA, BR, ABA, and JA via indirect competitive enzyme-linked immunosorbent assay (icELISA) according to Yang et al. (2001), Zhao et al. (2006), and Deng et al. (2008). Mouse monoclonal antigen and antibodies against free IAA, ZR, iPA, GA, BR, ABA, and JA were produced according to Weiler et al. (1981) at the Center of Crop Chemical Control, China Agricultural University, China. JA was derivatized into methyl jasmonate using JA in its free-acid form, and used for ELISA analysis. Anti-ZR antibody was used to detect ZR-type CKs, and the anti-iPA antibody was used to detect iP and iPR (iPA-type CKs). Calculation of the ELISA data was performed as described in Weiler et al. (1981). Percentage recoveries obtained using internal standards during extraction and analysis were all above 90%.

### Statistical analysis

A one-tailed *t*-test was used to compare the significance of differences in gene expression data from RT-PCR analysis. One-way ANOVA was used to compare the differences in phytohormone contents at different stages before pollination. All values are reported as means ± standard error (SE). Differences were considered significant at *P* < 0.05. To evaluate the relationship between phytohormone contents and expression levels of miRNAs and their targets, the qRT-PCR results and phytohormone levels of both the IFC and CFC ovaries were used for correlation analysis. All data analysis was performed using SPSS 19.0 (SPSS Inc., Chicago, IL, USA).

### Data access

The raw data of sRNA sequencing and degradome supporting the results of this study are available in the National Center for Biotechnology Informations (NCBI) Gene Expression Omnibus (GEO) database (http://www.ncbi.nlm.nih.gov/geo/), under accession GSE80998. The following link was created to allow review of record GSE80998: http://www.ncbi.nlm.nih.gov/geo/query/acc.cgi?token=khehakekjfmfjsd&acc=GSE80998

## Author contributions

CL participated in design of the study and wrote the manuscript. HL performed the experiments and data analysis, and wrote the draft manuscript. TP contributed to the data analysis and revised the manuscript. YW and QW participated in data analysis. JC and MZ participated in data analysis and field management. GT edited the manuscript and gave valuable advice on chart display. All authors read and approved the final manuscript.

### Conflict of interest statement

The authors declare that the research was conducted in the absence of any commercial or financial relationships that could be construed as a potential conflict of interest.
