# Supplementary material for: Development of Incompletely Fused Carpels in Maize Ovary Revealed by miRNA, Target Gene and Phytohormone Analysis
Source: Front Plant Sci. 2017 Apr 3;8:463. doi: 10.3389/fpls.2017.00463 (PMC5376576; doi:10.3389/fpls.2017.00463)
Supplement: Supplementary file 8 [file DataSheet1.DOCX]

**Development of incompletely fused carpelsin maize ovary revealed by miRNA, target gene and phytohormone analysis**

Hongping Li^1+^, Ting Peng^1+^, Yufeng Wu^2^, Xiuli Hu^1^,Qun Wang^1^,Moubiao Zhang^1^, Guiliang Tang^1^, Chaohai Li^1,^*

^1^Agronomy College, Collaborative Innovation Center of Henan Grain Crops, Henan Agricultural University, Zhengzhou 450002, P. R. China

^2^Bioinformatics Center, National Key Laboratory of Crop Genetics and Germplasm Enhancement, Nanjing Agricultural University, Nanjing 210095, P. R. China

*Corresponding author. Tel.:+86 371 63555629; E-mail address: lichaohai2016@sina.com

^+^These authors contributed equally to this work.

**Supplementary Figure S1**Reproducibility of small RNA sequencing data for two replicates of the IFC and CFC ovaries.Log_2_(NE+1) of the 162 miRNAs detected in at least one of the 4 sequenced samples were shown as scatter plots and were used for the Spearman correlation coefficient (SCC) analysis.The red diagonal line in each scatter plot denotes equal NE between two samples. NE, normalized expression; IFC, incompletely fused carpels; CFC, completely fused carpels.


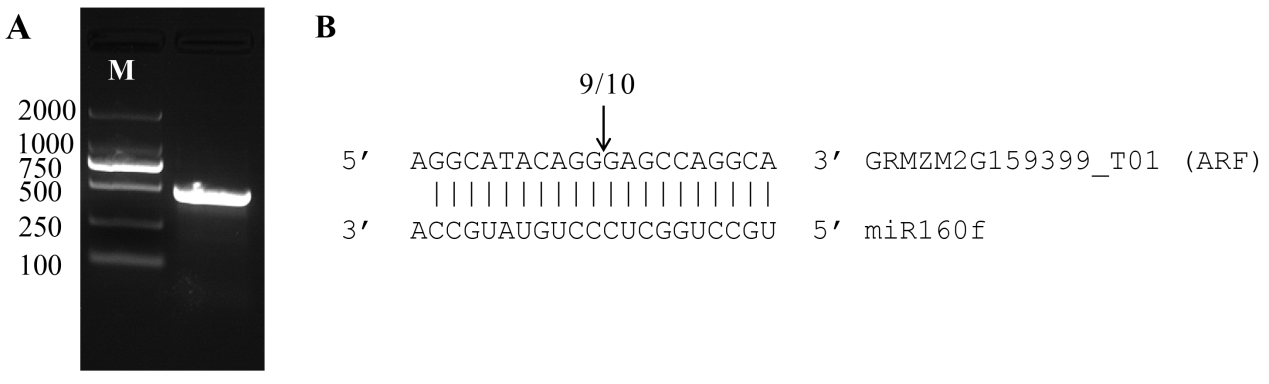


**Supplementary Figure S2** miR160f cleavage sites in GRMZM2G159399_T01 identified by RNA ligase-mediated 5’-RACE validation in IFC and CFC ovaries. The second round PCR product was showed on the ethidium bromide-stained agarose gel (A). The frequency of clones cleaved by miR160f was shown on the top of arrow (B). Watson-Crick pairing (vertical dashes) was indicated. Lane Mrepresents a DL2000 DNA ladder. ARF: auxin response factor; IFC, incompletely fused carpels; CFC, completely fused carpels.


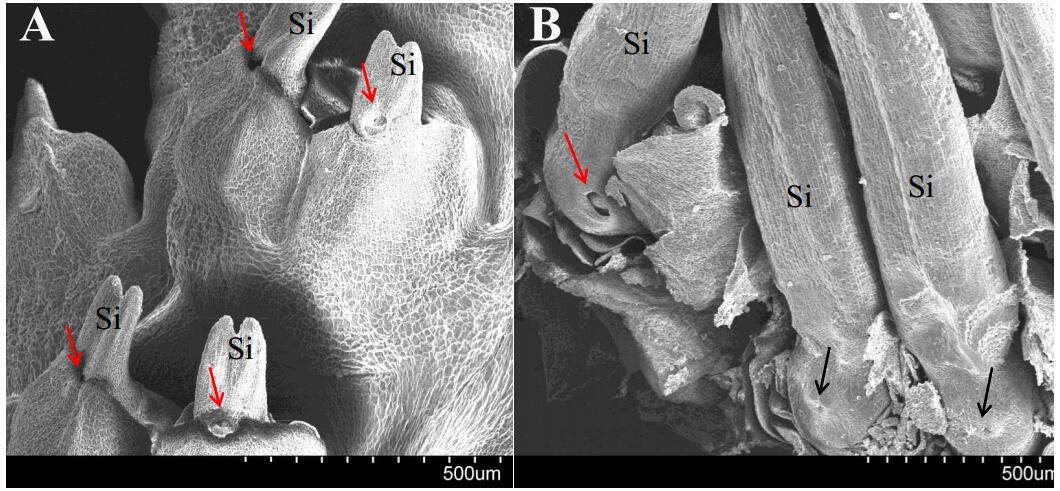


**Supplementary Figure S3** Scanning electron microscope observation of IFC ovary formation. A, carpel fusion was not yet finished during the initial stage of ovary formation, and the stylar canal was open. B, The initial observation of IFC ovary phenotype with open stylar canal, and CFC ovary had closed stylar canal. Red and dark arrows refer to compete and incomplete carpel fusion, respectively. Si, silk; IFC, incompletely fused carpels; CFC, completely fused carpels.

**Supplementary Table S1. Summary of known and predicated miRNAs**

| Groups | Total | |  | IFC | |  | CFC | |
| --- | --- | --- | --- | --- | --- | --- | --- | --- |
|  | Pre-miRNA | Unique miRNA |  | Pre-miRNA | Unique miRNA |  | Pre-miRNA | Unique miRNA |
| gp1a | 155 | 186 |  | 133 | 139 |  | 140 | 150 |
| gp1b | 6 | 9 |  | 4 | 5 |  | 4 | 5 |
| gp2 | 24 | 34 |  | 21 | 26 |  | 22 | 27 |
| gp4 | 521 | 877 |  | 480 | 641 |  | 501 | 707 |

gp1a: Reads map to maizemiRNAs/pre-miRNAs in miRbase and the pre-miRNAs further map to the genome & EST.gp1b: Reads map to selected (except for maize) miRNAs/pre-miRNAs in miRbase and the pre-miRNAs further map to the genome & EST.gp2: Reads map to miRNAs/pre-miRNAs in miRbase. The mapped pre-miRNAs do not map to the genome, but the reads (and of course the miRNAs of the pre-miRNAs) map to genome. The extended genome sequences from the genome loci may form hairpins.gp4: Reads do not map to pre-miRNAs in miRbase. But the reads map to genome & the extended genome sequences from genome may form hairpins.IFC, incompletely fused carpels;CFC, completely fused carpels.

**Supplementary Table S3 Summary of the degradome sequencing data inIFC and CFCovaries.**

| **Items** | **IFC** | | **CFC** | |
| --- | --- | --- | --- | --- |
| Raw reads | 16,915,044 |  | 16,680,835 |  |
| Reads < 15nt after removing 3 adaptor | 77,041 | (0.46%) | 76,318 | (0.46%) |
| Mappable reads | 16,838,003 | (99.54%) | 16,604,517 | (99.54%) |
| Unique raw reads | 6,061,835 |  | 6,257,951 |  |
| Unique reads < 15nt after removing 3 adaptor | 32,531 | (0.54%) | 33,367 | (0.53%) |
| Unique mappable reads | 6,029,304 | (99.46%) | 6,224,584 | (99.47%) |
| Transcript mapped reads | 12,835,530 | (75.88%) | 12,632,354 | (75.73%) |
| Unique transcript mapped reads | 4,843,581 | (79.90%) | 4,998,585 | (79.88%) |
| Number of input Transcript | 88,760 |  | 88,760 |  |
| Number of coverd transcript | 65,348 | (73.62%) | 65,533 | (73.83%) |
| IFC, incompletely fused carpels; CFC, completely fused carpels. | | |  |  |

**Supplementary Table S7 Summary of the differentially expressed novel miRNAs**

| miR_name | miR_seq | p_value | IFC (NE) | CFC (NE) | up/down |
| --- | --- | --- | --- | --- | --- |
| PC-5p-9534_356 | GAGTGATAGTCGTAGAGTAGCGTC | 0.000 | 86.58 | 201.42 | down |
| PC-3p-148558_19 | GTTTGTGGAATGGAATGAGTTGAT | 0.002 | 14.38 | 25.57 | down |
| PC-3p-41087_72 | CTTGAAAATCCGGAGGACC | 0.046 | 10.68 | 22.83 | down |
| PC-5p-189225_15 | AGGGGATTGAGGGGGCTATAATCC | 0.027 | 4.67 | 8.46 | down |
| PC-5p-3154_912 | AATTTCCCCCTCAATCCCCTCCAA | 0.000 | 964.40 | 549.40 | up |
| PC-5p-28564_110 | AAGGGCTGATTTGGTGACCCGGGA | 0.001 | 34.64 | 15.45 | up |
| PC-5p-15244_222 | AGACTTGCCCACTCAAATTTTCGC | 0.000 | 526.61 | 329.98 | up |
| PC-3p-57952_49 | ATCCCCTCCGGGATTGGTGTAACC | 0.002 | 30.49 | 13.14 | up |
| PC-3p-49411_59 | ATCCCCTCCGATCCTCCCGGGATC | 0.000 | 118.62 | 76.59 | up |
| PC-3p-1834_1333 | CCGTGGCTCCTGCTCCTGATG | 0.000 | 184.84 | 70.88 | up |

IFC, incompletely fused carpels; CFC, completely fused carpels; PC, predicted candidate; NE, normalized expression.

**Supplementary Figure S3Stem-loop structures of 8 selected novel miRNAs in maize ovary.**

**Supplementary Table S2 Summary of the 162 identified known miRNAs expressed in at least one of the 4 sequenced samples.** Expression values are presented as normalized data. Biological replicates are indicated as -1 and -2. (Excel file)

**Supplementary Table S4 miRNA target prediction results from maize IFC and CFCdegradome libraries.** TF, transcription factor; CFC, completely fused carpels; IFC, incompletely fused carpels; TPB, tags per billion. (Excel file)

**Supplementary Table S5 Target prediction results of the differentially expressed known miRNAs between maize IFC and CFCdegradome libraries.** TF, transcripton factor; CFC, completely fused carpels; IFC, incompletely fused carpels; TPB, tags per billion. (Excel file)

**Supplementary Table S6 Summary of the identified novelmiRNAsexpressed in at least one of the 4 sequenced samples.** Expression values are presented as normalized data. Biological replicates are indicated as -1 and -2. (Excel file)

**Supplementary Table S8 Target prediction results of the novel miRNAs from maize IFC and CFCdegradome libraries.** TF, transcripton factor; CFC, completely fused carpels; IFC, incompletely fused carpels; TPB, tags per billion. (Excel file)

**Supplementary Table S9 Target prediction results of the differentially expressed known miRNAs between maize IFC and CFCdegradome libraries.** TF, transcripton factor; CFC, completely fused carpels; IFC, incompletely fused carpels; TPB, tags per billion. (Excel file)

**Supplementary Table S10 Primer sequences of miRNAs and their targets used in qRT-PCR analysis.**(Excel file)
