# Supplementary material for: Development of Incompletely Fused Carpels in Maize Ovary Revealed by miRNA, Target Gene and Phytohormone Analysis
Source: Front Plant Sci. 2017 Apr 3;8:463. doi: 10.3389/fpls.2017.00463 (PMC5376576; doi:10.3389/fpls.2017.00463)
Supplement: Supplementary file 9 [file Image3.PDF]

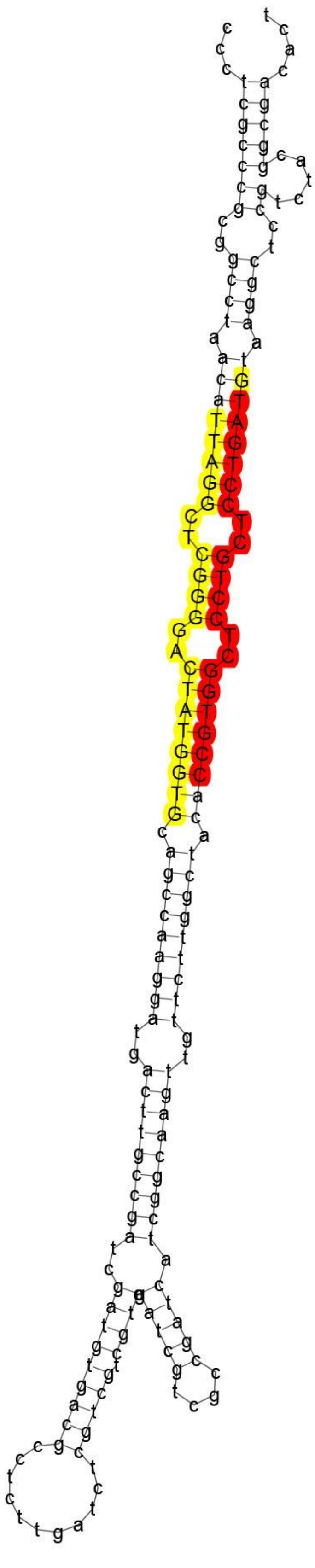

Secondary structure for PC-5p-2060_1226 (yellow) and PC-3p-1834_1333 (red)

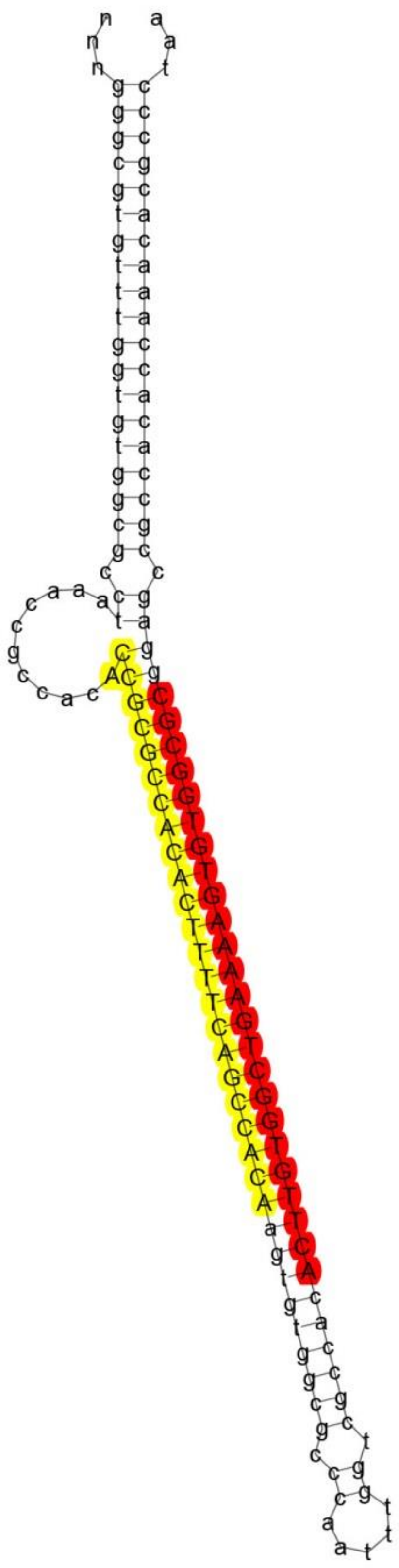

Secondary structure for PC-5p-95888_29 (yellow) and PC-3p-27812_113 (red)

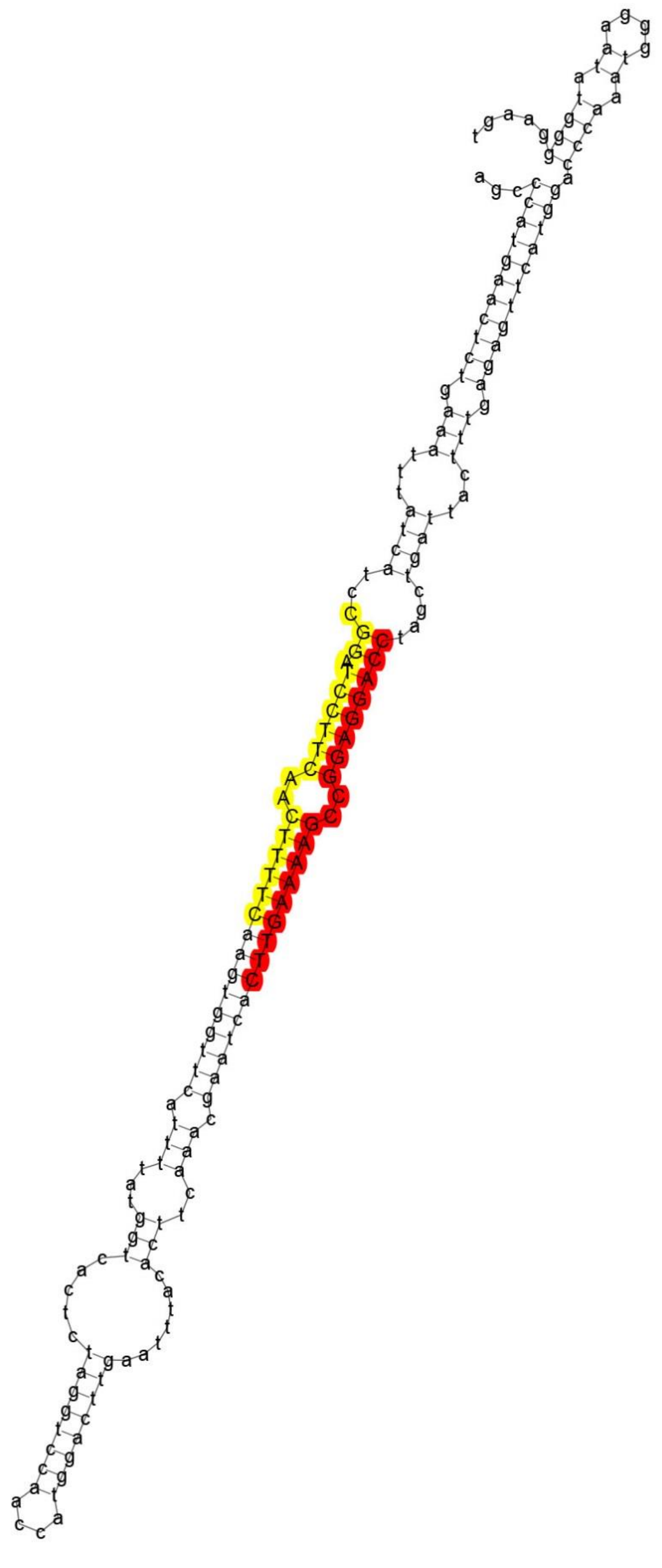

Secondary structure for PC-5p-3524575_1 (yellow) and PC-3p-41087_72 (red)

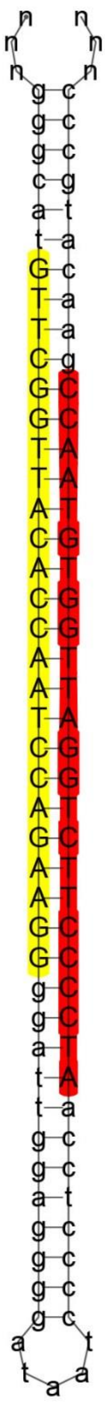

Secondary structure for PC-5p-539826_6 (yellow) and PC-3p-75737_37 (red

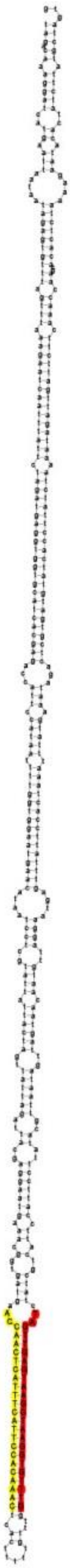

Secondary structure for PC-5p-5735457_1 (yellow) and PC-3p-148558_19 (red)

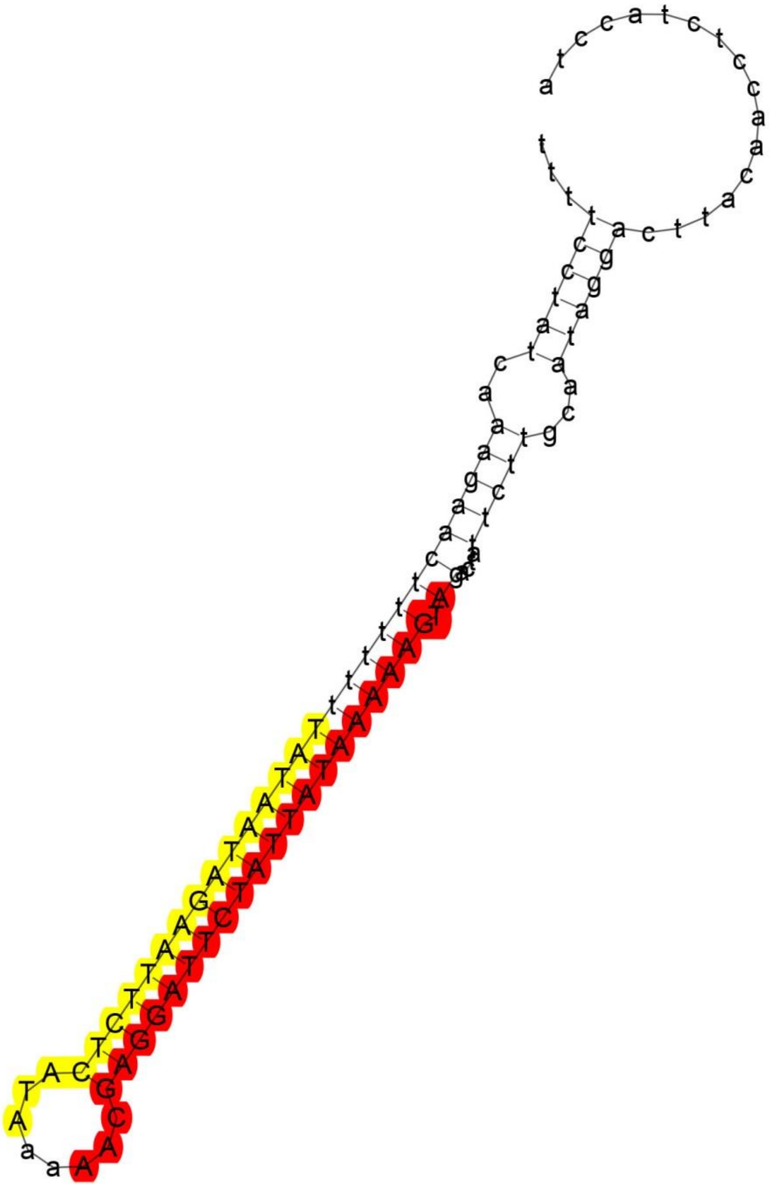

Secondary structure for PC-5p-1789121_2 (yellow) and PC-3p-274565_11 (red)

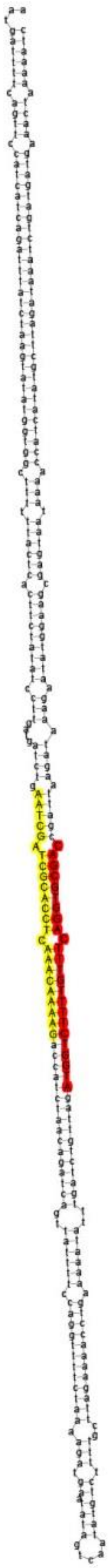

Secondary structure for PC-5p-48559_60 (yellow) and PC-3p-52873_55 (red)

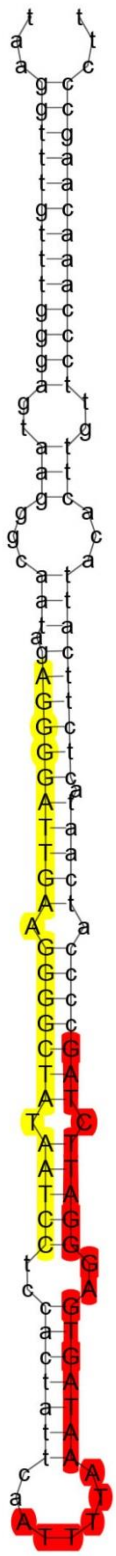

Secondary structure for PC-5p-189225_15 (yellow) and PC-3p-5948884_1 (red)
